# Familial Linkage between Neuropsychiatric Disorders and Intellectual Interests

**DOI:** 10.1371/journal.pone.0030405

**Published:** 2012-01-26

**Authors:** Benjamin C. Campbell, Samuel S.-H. Wang

**Affiliations:** 1 Neuroscience Institute, Princeton University, Princeton, New Jersey, United States of America; 2 Laboratory of Biological Modeling, Rockefeller University, New York, New York, United States of America; 3 Department of Molecular Biology, Princeton University, Princeton, New Jersey, United States of America; University of Sydney, Australia

## Abstract

From personality to neuropsychiatric disorders, individual differences in brain function are known to have a strong heritable component. Here we report that between close relatives, a variety of neuropsychiatric disorders covary strongly with intellectual interests. We surveyed an entire class of high-functioning young adults at an elite university for prospective major, familial incidence of neuropsychiatric disorders, and demographic and attitudinal questions. Students aspiring to technical majors (science/mathematics/engineering) were more likely than other students to report a sibling with an autism spectrum disorder (p = 0.037). Conversely, students interested in the humanities were more likely to report a family member with major depressive disorder (p = 8.8×10^−4^), bipolar disorder (p = 0.027), or substance abuse problems (p = 1.9×10^−6^). A combined PREdisposition for Subject MattEr (PRESUME) score based on these disorders was strongly predictive of subject matter interests (p = 9.6×10^−8^). Our results suggest that shared genetic (and perhaps environmental) factors may both predispose for heritable neuropsychiatric disorders and influence the development of intellectual interests.

## Introduction

A link between intellect and temperament has long been the subject of speculation. Aristotle claimed that “those who have become eminent in philosophy, politics, poetry, and the arts have all had tendencies toward melancholia”, while the physician Benjamin Rush noted a link between manic episodes and “talents for eloquence, poetry, music, and painting” [1]–[3]. Studies of the artistically inclined report linkage with familial depression [4], while among eminent and creative scientists, a lower incidence of affective disorders is found [5]. In the case of developmental disorders, a heightened prevalence of autism spectrum disorders (ASDs) has been found in the families of mathematicians, physicists, and engineers [6]–[8]. These threads of evidence suggest that intellectual interests might be broadly linked to neuropsychiatric disorders.

We had a unique opportunity to investigate such association in an entire defined population of high-functioning young adults, an incoming freshman class at a major private university. The students were ethnically and geographically diverse, and compared with the general population, academically motivated and relatively free to pursue their true interests [9]. This student body was biased towards middle and high socioeconomic status, groups with high levels of medical care and for whom familial neuropsychiatric issues are more likely to be detected and reported than in the general population. We obtained 1077 responses, which constitutes to our knowledge the largest cohort thus surveyed to date.

Consistent with prior findings, we noticed a relation between intended academic majors and ASDs. Looking for relations between other neuropsychiatric disorders and academic interest we also noted a heightened prevalence of bipolar disorder, major depressive disorder and substance abuse in the families of those pursuing the humanities. A composite score based on these four heritable disorders was strongly correlated with a student's intended academic major. Thus, familial risk toward a spectrum of psychopathologies can predict propensity toward technical versus humanist interests.

## Results

We surveyed the incoming class of 2014 at Princeton University about their intended academic major, familial incidence of neuropsychiatric disorders, and demographic variables. We received 1077 responses from 1313 students, a response rate of 82%. 527 respondents indicated a technical major (natural sciences, engineering, or mathematics), 394 indicated non-technical majors (245 in social sciences, 149 in humanities), and 156 students were undecided. A follow-up survey determined the mean number of siblings per student to be 1.5.

We began by looking for a previously reported relation between ASDs and technical interests [6]–[8]. Twenty-four freshmen (2.2%, 1 in 45) reported having a sibling with an ASD. This included 16 of 527 (3.0%, 1 in 33) aspiring technical majors compared with 4 of 394 (1.0%, 1 in 99) nontechnical students, for an odds ratio of 3.05 (χ^2^ = 4.33, p = 0.037). Thus the incidence of ASDs amongst siblings of technical majors was significantly higher than that of non-technical majors and roughly twice (after correction based on number of siblings) the US average of 1 in 160 [10].

**Figure 1:**
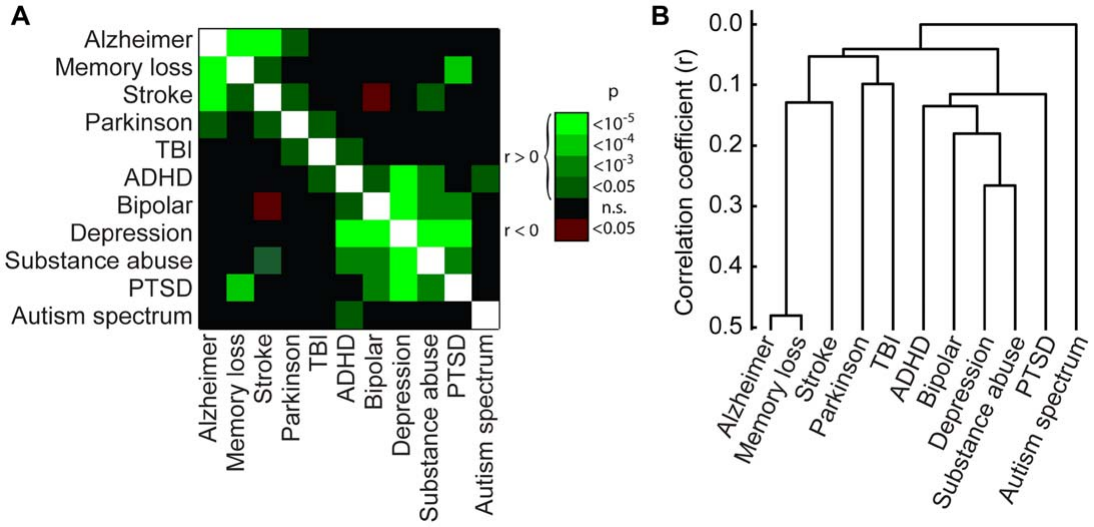
Familial co-occurrence of neuropsychiatric disorders. (a) Significant relations between disorders within families. Brightness represents p-value from χ^2^ test of independence, adjusted for multiple comparisons. (b) Correlation-based dendrogram calculated with a hierarchical agglomerative algorithm. Co-occurrence is familial except for ASD, for which sibling data were used.

We calculated relations between the reported familial incidence of surveyed disorders and other neurological conditions and two groupings became apparent (**Figure 1a**). One consisted of bipolar disorder, major depressive disorder, substance abuse, post-traumatic stress disorder (PTSD), and attention deficit hyperactivity disorder (ADHD). The other included Alzheimer's disease, memory loss, and stroke. On the basis of their correlations, we grouped these conditions with a hierarchical agglomerative algorithm (**Figure 1b**). Several of these relations reflect known comorbidities and familial aggregations [11]–[14].

We tested for correlations between individual disorders and intended major in a hypothesis-free manner. Significant associations were seen for bipolar disorder (p = 0.027), major depression (p = 8.8×10^−4^), and substance abuse (p = 1.9×10^−6^; all p-values adjusted for multiple comparisons) (**Table 1**). Each was represented most often among humanities majors, at intermediate rates in the social sciences, and least among technical majors. The two other disorders in this group were also most frequent in the families of prospective humanities majors but did not reach significance (ADHD, p = 0.10; PTSD, p = 0.31). From the second group, Alzheimer's approached significance but did not reach it (p = 0.069). The incidence of Parkinson's, stroke, and traumatic brain injury was roughly constant across subject matter interest.

**Table 1:** Relationships between neuropsychiatric disorders and intended academic major.

	Incidence of disorder	Odds ratioHumanities: Social sciences: Technical	p-value
Substance Abuse	167	3.19∶1.42∶1	1.9×10^−6^
Major Depression	150	2.48∶1.24∶1	8.8×10^−4^
Bipolar	51	2.76∶1.29∶1	0.027
Autism spectrum	20	0.66∶0.13∶1	0.037*
Alzheimer's	145	1.25∶1.71∶1	0.069
ADHD	85	2.00∶1.32∶1	0.10
PTSD	24	2.31∶1.18∶1	0.31
Memory loss	238	1.13∶1.35∶1	0.31
Parkinson's	53	0.97∶1.20∶1	0.89
Stroke	228	0.88∶1.01∶1	0.89
Traumatic brain injury	26	1.01∶1.24∶1	0.89

*ASD was tested as a prior hypothesis based on previous studies [6]–[8].

Incidence is defined as the number of reports of relatives (or siblings in the case of autism spectrum disorder, ASD) with a disorder among 921 responding students who specified an intended major.

**Figure 2:**
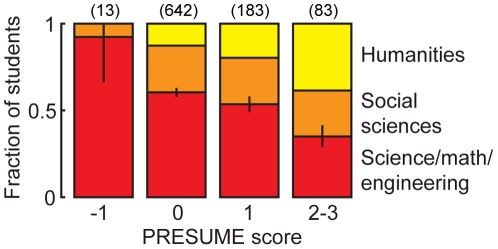
Relationship between subject matter preference and familial neuropsychiatric disorders. The PRESUME score was calculated as familial bipolar + depressive + substance abuse – ASD, with a maximum of one point per category. Error bars indicate 68% binomial proportion confidence intervals on the science/math/engineering fraction. The number in parenthesis indicates respondents in that category.

One traditional categorization of intellectual interests groups students into broad categories of science/technology/engineering/mathematical (STEM) disciplines, social sciences, and humanities. Information about a variety of familial disorders had the potential to be predictive of a student's position on that axis. We used the significant associations to define for each student a predisposition to subject matter (PRESUME) score by adding 1 point each for a report of bipolar disorder, depressive disorder, and substance abuse, and subtracting 1 point for a report of a sibling with ASD, resulting in an index that ranged from −1 to +3. The covariation of PRESUME with subject matter preference was strongly predictive of intended academic major (χ^2^ = 48.1, p = 9.6×10-8) (**Figure 2**). The overall technical:nontechnical odds ratio varied from 12∶1 (PRESUME = −1) to 1∶1.9 (PRESUME = +2 or +3), a 22-fold range (95% CI, 4-fold to >100-fold). Thus a combination of familial psychopathologies was predictive of a major intellectual dimension.

## Discussion

Our principal finding is that in a population of high-functioning young adults, intellectual interests are strongly related to the incidence of a variety of neuropsychiatric disorders in their families. Students interested in pursuing technical majors (science/mathematics/engineering) were significantly more likely to report a sibling with an autism spectrum disorder. Conversely, students interested in studying humanities were significantly more likely to report a family member with bipolar disorder, major depressive disorder, or substance abuse problems. A composite score based on these disorders was strongly predictive of an axis of student intellectual interest ranging from science/math/engineering to the humanities.

Neuropsychiatric disorders are interrelated and caused by combinations of genetic variants and environmental factors [15],[16]. The exact form of psychopathology expressed thus arises from a complex combination of alterations in cognitive, perceptual and affective capacity. Our survey identifies a dimension of normal intellectual variation that correlates with neuropsychiatric disorders. While we cannot rule out environmental influences for the relation reported here, the disorders contributing to the PRESUME index are moderately to strongly heritable [17], [18], suggesting that their genetic determinants may influence the normal development of intellectual interests. Psychopathological traits, temperament, and interests all begin to develop early in life [19], consistent with these phenotypes sharing overlapping developmental genes. Our findings suggest that subject matter interest is another such heritable trait.

It has previously been reported that a higher incidence of ASDs occurs in the families of engineers and scientists [6], [20] and students studying mathematics, physics, and engineering [7], [8]. Our results provide further support for a such a relation between ASDs and technical subjects. It has been suggested that autism represents an extreme manifestation of a “systemizing” nature [21]. Since ASDs have complex inheritance, shared genetic variation between close relatives might establish a continuous phenotype which in milder forms confers interest or benefits in understanding highly structured fields, and in extreme forms is dysfunctional.

Similarly, affective disorders may represent an extreme phenotype of emotional lability that, in milder forms, is commensurate with interest in the humanities. Prior research on familial linkage with psychopathology has focused on both subject interests and creativity. One study noted an increased incidence of bipolar disorder in literature students [7]. A heightened incidence of affective disorders has also been reported in artists, based on posthumous biographical studies [3], [22] and studies of living writers and their relatives [4], [23]. However, studies including eminent scientists have noted that the incidence of these disorders is decreased [5], [24]. Scientific creativity differs from creativity in the arts, requiring a strong element of logical deduction [25]. Our report of differing psychopathological patterns in the “two cultures” [26] suggests that any linkage between psychopathology and creativity is likely to differ depending on whether one is interested primarily in arts or sciences.

Our findings do not speak to the respective contributions of genes and environmental influences in shaping intellectual interests. Environmental influences can take many forms, including the experience of growing up with the presence of mental illness within a family. Experience can shape the developing brain and reinforce preexisting tendencies or lead in unexpected directions. Future studies are needed to separate the influences of genes and shared environment on subject matter interests. In any case, our study reinforces the notion that psychopathology is an extreme form of mental function related to normal human phenotypic variation.

## Materials and Methods

We performed an online survey (using Qualtrics software) to assess attitudes and knowledge about science, familial incidence of neuropsychiatric disorders, demographic variables, and intended academic major. The survey also tested students for synesthesia and prosopagnosia. The web address of the survey was sent exclusively to the Princeton University entering Class of 2014, and was available from July 23, 2010 to September 1, 2010. After this date the URL was inadvertently made public and further responses were disregarded. Of the replies received, responses were removed if the IP address was duplicated (typically in two consecutive sessions) or if responses were sparse. Student identity was not collected. A summary of the survey questions and results can be found at http://synapse.princeton.edu/freshman-survey-report.pdf.

To assess subject matter interest, students were asked “Based on your current interests, what might be your likely major?” with the following options: Physical sciences or math, other natural sciences, engineering, humanities, social sciences, specific major in mind (specify), and undecided. Specific majors were classified in their parent category; multiple answers spanning categories were classified as undecided. For neuropsychiatric disorders, students were asked “Among your immediate family (parents, siblings, and yourself) and grandparents, which of the following major events have occurred? Check all that apply”, followed by a checklist of 17 disorders divided into two parts. Finally, a standalone question was asked: “Do you have a sibling with autism spectrum disorder (ASD)? ASD includes autism, Asperger's, and pervasive developmental disorder not otherwise specified.” To obtain the average number of siblings, a separate in-person survey was done with ∼90% response rate (n = 131) in classes in which technical and nontechnical students were enrolled.

Four disorders with low response frequency were not included in the analysis: Huntington's disease (n = 1), phantom limb syndrome (n = 4), epilepsy (n = 18), and schizophrenia (n = 18). Additionally, the disorder of “trouble recognizing faces,” which was intended to gauge prosopagnosia, was found to depend strongly on memory loss (χ^2^ = 138.2, p = 6.7×10^−32^) and was discarded from the analysis. This left eleven neuropsychiatric variables (including ASD). To display the relations between these variables (Figure 1a), p-values were calculated based on a χ^2^ test of independence and adjusted for multiple comparisons to control for false discovery rate using the Benjamini-Hochberg procedure [27]. Agglomerative hierarchical clustering (Figure 2b) was performed using the MATLAB Statistics toolbox, with pairwise similarities between disorders were calculated based on the sample correlation coefficient r and disorders clustered by an average-linked measure of the distance metric d = (1-r)/2.

Since two questions were asked concerning ASDs, a conservative approach was taken by scoring responses as positive only if both questions were answered in the affirmative. Seven respondents indicated a sibling with an ASD without indicating a relative with an ASD (three technical majors, one non-technical major, three undecided), an inconsistency that may have arisen because Asperger's and pervasive developmental disorder not otherwise specified were listed explicitly in the sibling question but not in the familial question. Inclusion of these respondents (for a total of n = 31 reporting siblings with an ASD, or 1 in 35) increased the statistical significance of the result (χ^2^ = 4.85, p = 0.028).

Since a relationship between ASDs and technical majors (math/science/engineering) has been previously reported [7], [8], this relation was established by a chi-square test of independence comparing technical majors vs. non-technical majors. For other disorders, a relation with intended major was examined in a hypothesis-free manner. Each disorder was compared between technical majors, social sciences, and humanities, by a chi-square test of independence, with p-values (Table 1) adjusted to control for false discovery rate.

### Ethics Statement

The survey was originally conducted for educational purposes. Retrospective approval to analyze anonymous data was granted by the Princeton University Institutional Review Board.
